# Association Between HbA1c Levels and the Severity of Diabetic Retinopathy

**DOI:** 10.7759/cureus.76395

**Published:** 2024-12-25

**Authors:** Nayef Alswaina

**Affiliations:** 1 Department of Ophthalmology, College of Medicine, Qassim University, Kingdom of Saudi Arabia, Buraidah, SAU

**Keywords:** diabetes complications, diabetic retinopathy, glycemic control, saudi arabia, type 2 diabetes

## Abstract

Background: Diabetic retinopathy (DR) is a significant microvascular complication of diabetes mellitus (DM), contributing to visual impairment and blindness worldwide. Understanding the factors associated with the severity of DR is crucial for effective prevention and management. This study aimed to explore the association between hemoglobin A1c (HbA1c) level and other parameters with different stages of DR.

Methodology: A retrospective cross-sectional study was conducted on patients diagnosed with DR between March and September 2024. Patients with type 2 diabetes who underwent fundus examinations and had HbA1c measurements were analyzed. Data on demographic and clinical parameters, including age, gender, duration of diabetes, glycemic control, anti-diabetic medications, body mass index (BMI), and diabetes-related complications, were collected. The severity of DR was classified into mild, moderate, and severe non-proliferative diabetic retinopathy (NPDR) and proliferative diabetic retinopathy (PDR). Statistical analyses included chi-square tests, independent t-tests, and analysis of variance (ANOVA), with *P*-values < 0.05 considered significant.

Results: A total of 106 participants were included, with a mean age of 57.3 years. PDR was present in 27 patients (25.5%), while 43 patients (40.6%) had mild NPDR. Very poor glycemic control (HbA1c > 9%) was noted in 36 patients (34%). Insulin use was significantly associated with higher DR severity (*P *= 0.039), while macular edema showed a strong association with advanced DR stages (*P *= 0.003). No significant associations were found between DR severity and neuropathy or nephropathy.

Conclusions: The study highlights that uncontrolled glycemic level, longer diabetes duration, and the presence of macular edema are key factors associated with the severity of DR. Insulin use may indicate a higher risk of severe DR, emphasizing the need for comprehensive diabetes management and regular ophthalmologic evaluations. These findings underscore the importance of targeted interventions to prevent vision-threatening complications in diabetic patients.

## Introduction

Diabetes mellitus (DM) is a long-term metabolic condition characterized by hyperglycemia due to an absolute or relative lack of insulin. Globally, about 463 million individuals are affected with DM with a prevalence rate of 9.3%, which is projected to increase to 10.2% by 2030 [1,2]. In Saudi Arabia, the prevalence of DM is much higher, reaching 30% in 2011, and is becoming increasingly common [3,4]. Chronic hyperglycemia can lead to serious, life-threatening conditions, including cardiovascular complications, retinopathy, nephropathy, and neuropathy [5]. Therefore, understanding the relationship between DM and its complications by early detection and treatment is crucial in preventing their progression, which results in the reduction of morbidity and mortality among those patients.

Diabetic retinopathy (DR) is well well-known sight-threatening microvascular complication of DM. It is the most common cause of blindness among individuals aged between 20 and 74 years old. In Saudi Arabia, a recent study showed that the prevalence of DR among diabetics was a range between 30% and 36% [6-8]. The progression of DR in patients with diabetes is influenced by various risk factors, including modifiable ones such as glycemic levels, blood pressure, lipid profiles, and smoking, as well as nonmodifiable factors like the duration of DM, patient age, and genetic predisposition [5,9]. Hemoglobin A1c (HbA1c) has low variability and was found to be a good indicator of long-term control of glycemic level in DM patients and could be correlated with the severity of DM [10,11]. This study aims to investigate the association between serum HbA1c levels and the severity of DR in type 2 diabetic patients so that progression of the condition can be predicted and early intervention can be established.

## Materials and methods

This retrospective, analytical cross-sectional study was conducted in the outpatient clinics of Qassim University Medical City after obtaining ethical approval from the Research Ethics Committee of the institute (approval IRB-24-12-05). Medical records of patients diagnosed with DR who were presented to the ophthalmology clinic between March and September 2024 were retrospectively reviewed. Subjects with complete medical records were included in the study. The inclusion criteria encompassed type 2 diabetic patients aged 18 years or older, who had undergone a fundus eye examination during an ophthalmology visit and had a recorded HbA1c laboratory result. Medical records that lacked any of these criteria or had incomplete demographic or clinical data were excluded.

Data collection involved retrieving demographic and clinical parameters. Demographic data included age and gender, while clinical data covered variables such as the duration of diabetes, age at onset of diabetes, glycemic control (HbA1c level classified as good control if <7%, poor control if 7%-9%, and very poor control if >9%), the use of anti-diabetic drugs, the presence of dyslipidemia, obesity (categorized based on BMI as overweight: 25.0-29.9 kg/m², obese: 30.0-39.9 kg/m², and morbidly obese: ≥40.0 kg/m²), blood pressure status (normotensive or hypertensive requiring antihypertensive drugs), and diabetes-related complications such as neuropathy and nephropathy. The severity of DR was classified into mild non-proliferative diabetic retinopathy (NPDR), moderate NPDR, severe NPDR, and proliferative diabetic retinopathy (PDR) according to the International Clinical Diabetic Retinopathy Disease Severity Scale.

A pilot study was conducted among DR patients at Qassim University Hospital to evaluate the logistics of data collection, ensure the clarity and appropriateness of the data collection tools, and estimate the time required for the collection process. The findings from the pilot study guided refinements to the data collection methodology.

Data management and statistical analysis were conducted using SPSS software. Frequencies and percentages were calculated for all studied variables. Cross-tabulation analyses were performed using the chi-square test, while comparisons between groups were analyzed using independent t-tests and analysis of variance (ANOVA) tests. A *P*-value of <0.05 was considered statistically significant. All data were anonymized to maintain confidentiality and were used exclusively for research purposes.

## Results

A total of 106 participants were included in the study. Half of the patients were older than 60 years old, with a total mean of the sample of 60.6 (standard deviation [SD] = 11.76) years. There was a slight predominance of males (57, 53.8%) compared to females (49, 46.2%). Regarding the duration of diabetes, 31 of the participants (29.3%) had diabetes for 1-10 years, 44 (41.4%) for 11-20 years, and 31 (29.3%) for more than 20 years. In addition, most of the participants had poor and very poor control HbA1c (52, 49.0%) and (36, 34%), respectively, while 18 (17%) had good control HbA1c. Oral hypoglycemic agents were used by 55 (51.9%) of participants, while 51 (48.1%) were on insulin alone or in combination with other drugs. Regarding BMI, 46 participants (43.4%) were classified as obese, 37 (34.9%) as overweight, and 23 (21.7%) as having a normal weight. Hypertension was observed in 57 (53.8%) of participants, while 49 (46.2%) were normotensive (Table 1).

**Table 1:** Demographic factors of the participants. DM, diabetes mellitus; BMI, body mass index

Demographic factors		*n* (%)
Age (years)	Mean (SD)	60.6 (11.76)
	<60	53 (50.0%)
	>60	53 (50.0%)
Gender	Male	57 (53.8%)
	Female	49 (46.2%)
Duration of DM	1-10	31 (29.3%)
	11-20	44 (41.5%)
	>20	31 (29.2%)
HbA1c	Good control	18 (17.0%)
	Poor control	52 (49.0%)
	Very poor control	36 (34.0%)
Anti-diabetic medication	Oral hypoglycemic agent only	55 (51.9%)
	Insulin with or without drugs	51 (48.1%)
BMI	Normal weight	23 (21.7%)
	Overweight	37 (34.9%)
	Obese	46 (43.4%)
Blood pressure	Normotensive (<140/90 mmHg)	49 (46.2%)
	Hypertensive (receive antihypertensive treatment)	57 (53.8%)

Neuropathy was present in 13 patients (12.3%), whereas nephropathy was observed in 17 patients (16.0%). Macular edema was present in 42 (39.6%) participants, whereas 64 (60.4%) did not have this condition. Regarding the severity of DR, 43 (40.6%) participants had mild NPDR, 29 (27.4%) had moderate NPDR, 7 (6.6%) had severe NPDR, and 27 (25.5%) had proliferative DR (Table 2).

**Table 2:** Prevalence of diabetic complications. NPDR, non-proliferative diabetic retinopathy; DR, diabetic retinopathy

Complications		*n* (%)
Neuropathy	Absent	93 (87.7%)
	Present	13 (12.3%)
Nephropathy	Absent	89 (84.0%)
	Present	17 (16.0%)
Macular edema	Absent	64 (60.4%)
	Present	42 (39.6%)
Retinopathy	Mild NPDR	43 (40.6%)
	Moderate NPDR	29 (27.4%)
	Severe NPDR	7 (6.6%)
	Proliferative DR	27 (25.5%)

The severity of DR was analyzed concerning demographic and clinical variables. Although there was no statistically significant association between age and retinopathy severity, proliferative DR was more frequent in participants aged <60 years. Gender was not significantly associated with DR severity, with similar distributions among males and females. A longer duration of diabetes was linked to higher rates of proliferative DR, particularly among those with diabetes for 11-20 years, though the association was not statistically significant. Participants with very poor control HbA1c levels (HbA1c > 9%) had the highest proportion of proliferative DR, but this association was also not statistically significant. Anti-diabetic medication showed a significant association with DR severity (*P *= 0.039), where participants on insulin had a higher likelihood of severe retinopathy compared to those on oral hypoglycemic agents. BMI and blood pressure were not significantly associated with retinopathy severity, though hypertensive participants exhibited slightly higher rates of proliferative DR (Table 3).

**Table 3:** Association between severity of retinopathy and other variables. *Significant at a *P*-value < 0.05. NPDR, non-proliferative diabetic retinopathy; DR, diabetic retinopathy; DM, diabetes mellitus; HbA1c, hemoglobin A1c; BMI, body mass index

Variables		Mild NPDR		Moderate NPDR		Severe NPDR		Proliferative DR		*P*-value
		n	%	n	%	n	%	n	%	
Age (years)	<60	21	39.6%	12	22.6%	5	9.4%	15	28.4%	0.584
	>60	22	41.5%	17	32.1%	2	3.8%	12	22.6%	
Gender	Male	25	43.9%	14	24.6%	4	7.0%	14	24.6%	0.860
	Female	18	36.7%	15	30.6%	3	6.1%	13	26.5%	
Duration of DM	1-5	15	48.4%	7	22.6%	3	9.7%	6	19.3%	0.903
	11-20	17	38.6%	11	25.0%	3	6.8%	13	29.5%	
	>20	11	35.5%	11	35.5%	1	3.2%	8	25.8%	
HbA1c	Good control	10	55.6%	4	22.2%	0	0.0%	4	22.2%	0.647
	Poor control	19	36.5%	16	30.8%	5	9.6%	12	23.1%	
	Very poor control	14	38.9%	9	25.0%	2	5.6%	11	30.6%	
Anti-diabetic medication	Oral hypoglycemic agent only	29	52.7%	10	18.2%	4	7.3%	12	21.8%	0.039*
	Insulin with or without drug	14	27.5%	19	37.3%	3	5.9%	15	29.4%	
BMI	Normal weight	6	26.1%	6	26.1%	3	13.0%	8	34.8%	0.451
	Overweight	14	37.8%	12	32.4%	2	5.4%	9	24.3%	
	Obese	23	50.0%	11	23.9%	2	4.3%	10	21.7%	
Blood pressure	Normotensive (<140/90 mmHg )	18	36.7%	18	36.7%	4	8.2%	9	18.4%	0.145
	Hypertensive (receive antihypertensive treatment )	25	43.9%	11	19.3%	3	5.3%	18	31.6%	

The presence of neuropathy and nephropathy did not show significant associations with DR severity. However, the presence of macular edema was statistically significantly associated with DR severity, which showed that patients with more severe types of DR had a higher percentage of developing macular edema (*P *= 0.003) (Table 4).

**Table 4:** Association between retinopathy severity and presence of other complications. *Significant at *P*-value < 0.05. NPDR, non-proliferative diabetic retinopathy; DR, diabetic retinopathy

Complications		Retinopathy								*P*-value
		Mild NPDR		Severe NPDR		Severe NPDR		Proliferative DR		
		n	%	n	%	n	%	n	%	
Neuropathy	Absent	40	43.0%	24	25.8%	6	6.5%	23	24.7%	0.578
	Present	3	23.1%	5	38.5%	1	7.7%	4	30.8%	
Nephropathy	Absent	39	43.8%	22	24.7%	6	6.7%	22	24.7%	0.391
	Present	4	23.5%	7	41.2%	1	5.9%	5	29.4%	
Macular edema	Absent	34	53.1%	15	23.4%	1	1.6%	14	21.9%	0.003*
	Present	9	21.4%	14	33.3%	6	14.3%	13	31.0%	

## Discussion

This study investigated the demographic and clinical factors associated with the severity of DR among patients with type 2 diabetes at Qassim University Medical City. The findings highlight several critical factors contributing to the progression and severity of DR and underscore the importance of managing diabetes-related complications to prevent advanced DR stages.

Demographic factors and DR severity

The results revealed that older age groups (≥60 years) constituted half of the study population, with most DR cases falling within mild or moderate NPDR categories. Although no statistically significant association was found between age and DR severity, the slightly higher prevalence of PDR among younger participants (<60 years) suggests that younger patients with uncontrolled diabetes or longer disease duration may be at greater risk of severe complications. Similar findings have been reported in previous studies, which highlighted a complex interplay between age, duration of diabetes, and glycemic control in determining DR severity [12-15].

Glycemic control and DR severity

Very poor glycemic control, reflected by elevated HbA1c levels (>9%), was associated with a higher prevalence of PDR, although this did not reach statistical significance in our study. This is in agreement with the results of the previous study of Almutairi et al. However, many studies showed a significant association between HbA1c level and severity of DR [16]. Prolonged hyperglycemia leads to microvascular damage, contributing to DR progression [17,18]. Studies have consistently demonstrated that maintaining HbA1c levels below 7% reduces the risk of DR progression [19,20]. The predominance of participants with HbA1c levels > 7% and the high proportion with poorly controlled diabetes highlight the need for intensified glycemic management strategies to mitigate DR progression.

Duration of diabetes and DR severity

A longer duration of diabetes was positively associated with DR severity, with the highest rates of PDR observed in participants with diabetes lasting 11-20 years. Chronic hyperglycemia over time increases the risk of microvascular complications, including DR [21]. This aligns with findings from a previous study, which demonstrated that DR risk increases with diabetes duration regardless of other risk factors [22]. Early screening and consistent follow-up for patients with longer diabetes durations are, therefore, critical to detect and manage DR in its early stages.

Role of anti-diabetic medications

The study identified a statistically significant association between the use of anti-diabetic medications and DR severity. Participants using insulin, either alone or in combination with oral hypoglycemic agents, exhibited higher rates of moderate-to-severe DR compared to those on oral agents alone. This finding may reflect the fact that insulin use is more common in patients with long-standing or poorly controlled diabetes, conditions inherently associated with increased DR severity [23,24]. However, it underscores the importance of comprehensive management strategies that combine pharmacological interventions with lifestyle modifications to optimize outcomes.

Complications and DR severity

Macular edema showed a significant association with severe NPDR and PDR. This aligns with prior research that identified macular edema as a critical determinant of visual impairment and advanced DR stages [25-27]. Surprisingly, neuropathy and nephropathy were not significantly associated with DR severity in this study, which may be due to the small number of participants with these complications. Nonetheless, the interplay between these complications warrants further exploration, as previous studies have established their coexistence with DR in poorly controlled diabetes [28,29].

Clinical implications

The findings of this study emphasize the need for early screening and timely management of DR, particularly in patients with poor glycemic control, long-standing diabetes, and those receiving insulin therapy. Regular fundus examinations and targeted interventions to manage glycemic levels, blood pressure, and lipid profiles are essential for preventing progression to advanced DR stages. Additionally, addressing macular edema through appropriate therapies, such as intravitreal anti-vascular endothelial growth factor (VEGF) injections, may help reduce the burden of vision-threatening DR [30].

Strengths and limitations

This study adds to the existing literature by providing insights into DR and its associated factors in a specific population within Saudi Arabia. However, it is limited by its retrospective design and reliance on medical records, which may introduce selection bias and data incompleteness. Future prospective studies with larger sample sizes are recommended to validate these findings and explore other potential risk factors for DR progression.

## Conclusions

The study underscores the importance of early identification and management of risk factors associated with DR severity such as glycemic control and diabetes duration. Comprehensive diabetes care strategies, including patient education and regular ophthalmologic evaluations, are essential for mitigating the burden of DR and improving patient outcomes. Further research is needed to evaluate interventions that can effectively reduce DR progression in diverse patient populations.
